# Discontinuation of antiviral prophylaxis correlates with high prevalence of hepatitis B virus (HBV) reactivation in rheumatoid arthritis patients with HBV carrier state: a real-world clinical practice

**DOI:** 10.1186/1471-2474-15-449

**Published:** 2014-12-22

**Authors:** Ying-Qian Mo, An-Qi Liang, Jian-Da Ma, Le-Feng Chen, Dong-Hui Zheng, H Ralph Schumacher, Lie Dai

**Affiliations:** Department of Rheumatology, Sun Yat-sen Memorial Hospital, Sun Yat-Sen University, 107# Yan Jiang West Road, Guangzhou, 510120 People’s Republic of China; Division of Rheumatology, VA Medical Center, University of Pennsylvania, University &, Woodland Aves, Philadelphia, PA USA

**Keywords:** Rheumatoid arthritis, Hepatitis B virus, Disease-modifying antirheumatic drugs

## Abstract

**Background:**

To investigate the risk of hepatitis B virus (HBV) reactivation in rheumatoid arthritis (RA) patients with HBV carrier state during treatment of disease-modifying antirheumatic drugs (DMARDs) and the use of antiviral prophylaxis in real-world clinical practice.

**Methods:**

Consecutive RA patients with HBV carrier state were included. Clinical data including liver evaluation, HBV infection evaluation and the use of antiviral prophylaxis were recorded.

**Results:**

Fifty-three RA patients with HBV carrier state were screened and 36 patients were qualified for analysis. Thirty-six percentage of patients developed HBV reactivation and 17% developed HBV hepatitis together with reactivation, one of which developed decompensate cirrhosis. Only 50% of patients accepted lamivudine although all patients were recommended antiviral prophylaxis with entecavir or tenofovir and only 31% continued during DMARDs therapy. Seventy-one percentage of patients who discontinued antiviral prophylaxis developed HBV reactivation 3 ~ 21 months after discontinuation. Logistic regression analyses showed discontinuation of antiviral prophylaxis (OR: 66, p = 0.027), leflunomide (OR: 64, p = 0.011) and past history of hepatitis (OR: 56, p = 0.013) were risk factors of HBV reactivation. Past history of hepatitis (OR: 10, p = 0.021) was also risk factor of HBV hepatitis together with reactivation.

**Conclusion:**

Our results suggest poor patient acceptance and discontinuation of antiviral prophylaxis should not be ignored for Chinese RA patients with HBV carrier state in real-world clinical practice. Discontinuation of antiviral prophylaxis, past history of hepatitis and LEF might increase risk of HBV reactivation for RA patients with HBV carrier state during DMARDs therapy.

**Electronic supplementary material:**

The online version of this article (doi:10.1186/1471-2474-15-449) contains supplementary material, which is available to authorized users.

## Background

Chronic hepatitis B virus (HBV) infection is defined as the presence of positive surface antigen of HBV (HBsAg) more than 6 months and generally classified into HBV hepatitis with fluctuant alanine amino-transferase (ALT) and HBV carriers state with persistent normal ALT. Chronic HBV infection is a global healthcare problem affecting more than 350 million people around the world with potential poor prognosis of cirrhosis, hepatocarcinoma or death [1, 2]. Our previous study reported the prevalence of chronic HBV infection among Chinese rheumatoid arthritis (RA) patients was 11.2%, similar with the prevalence of the age-matched general Chinese population [1, 3]. It is estimated 300,000 ~ 600,000 Chinese RA patients with chronic HBV infection.

The prevalence of HBV reactivation was estimated 50% among patients with hematology-oncology diseases during immunosuppressive therapy and it may lead to severe outcomes including hepatitis, acute liver failure, cirrhosis or death in 5% ~ 30% of patients [4]. International associations for the study of liver disease recommended antiviral prophylaxis should be used before immunosuppressive therapy and continued minimal 6 ~ 12 months after suspension of immunosuppressant for chronic HBV infection patients [5–7], based on clinical evidences derived from hematology-oncology field. However, there’re great differences of host immune status, the types and intensity of immunosuppressant between RA patients and hematology-oncology patients.

Recommendations for disease-modifying antirheumatic drugs (DMARDs) from American College of Rheumatology (ACR) in 2008 suggested that minocycline, sulfasalazine (SSZ) under antiviral prophylaxis and hydroxychloroquine (HCQ) could be used for RA patients with HBV carrier state and liver function of Child-Pugh class A (a scoring system for chronic liver disease), while methotrexate (MTX) and leflunomide (LEF) were contraindicated [8]. ACR 2012 update recommended that biologic DMARDs should also be used with antiviral prophylaxis in these patients [9]. That is to say, long-term antiviral prophylaxis should be used for RA patients with HBV carrier state due to long-term DMARDs treatment. However, in real-world clinical practice, antiviral prophylaxis may not be continued even not accepted due to high economic burden, poor patient compliance or efficacy/safety of antiviral drugs. To explore the risk of HBV reactivation in RA patients with HBV carrier state during DMARDs therapy and the use of antiviral prophylaxis in real-world clinical practice, here we reported our single center results.

## Methods

### Patients

Consecutive and hospitalized RA patients from July 2007 to September 2013 at department of Rheumatology, Sun Yat-Sen Memorial Hospital, Sun Yat-Sen University were screened. All RA patients fulfilled 1987 ACR revised criteria or 2010 ACR/European League Against Rheumatism (EULAR) criteria. RA Patients with HBV carrier state who had positive HBsAg, normal ALT ≥ 6 months and normal total billirubin (TBiL) were included. Patients with HBV hepatitis or other types of viral hepatitis, autoimmune hepatitis, drug-induced hepatitis, cirrhosis or hepatocarcinoma were excluded. All patients gave written informed consent for this study, which was approved by the Ethics Committee of Sun Yat-Sen Memorial Hospital.

### Study design

Clinical data such as demographic characteristics, RA disease activity evaluation, liver evaluation, HBV infection evaluation and therapeutic regimens were recorded during follow-up period without interference with physicians’ therapeutic strategies. ***Liver evaluation*** included serum ALT, TBiL and if necessary, liver ultrasonography. ***HBV infection evaluation*** included serum HBV-DNA and HBV serological markers including HBsAg and its antibody (HBsAb), antigen e of HBV (HBeAg) and its antibody (HBeAb), antibody to HBV core antigen (HBcAb). Serum HBV-DNA was detected by quantitative real-time PCR by fluorogenic probe method with a lower limit of detection of 10^3^copies/mL. HBV serological markers were qualitatively detected by ELISA.

### Outcomes

The primary outcome was HBV reactivation, which was defined as a 10-fold rise in HBV-DNA compared to baseline or a switch from undetectable to detectable, and/or HBeAg seroconversion from negative to positive [4]. The secondary outcome was HBV hepatitis defined as ALT > 80U/L after reactivation, with or without icterus [10].

### Statistical analysis

Statistical analysis was performed with SPSS for Windows 13.0 (SPSS Inc., Chicago, IL, USA). The non-parametric Mann–Whitney U test or Fisher’s exact probabilities test were used for between-group comparison. Survival curve by Kaplan–Meier method and log-rank test was used to estimate the occurrence time of HBV reactivation. Step-forward logistic regression analysis was used to find out the risk factors of HBV reactivation and the following HBV hepatitis, counting odds ratio (OR) and its 95% of confidence interval (CI). A p-value of less than 0.05 was considered to be significant.

## Results

### Baseline characteristics of the study patients

Four hundred and ninety-six consecutive and hospitalized RA patients were screened. Three patients with HCV hepatitis, one patient overlapping with autoimmune hepatitis and two patients with drug-induced hepatitis were excluded. None of these six patients had positive HBsAg. Seven patients with HBV hepatitis were not included either.

**Figure 1:**
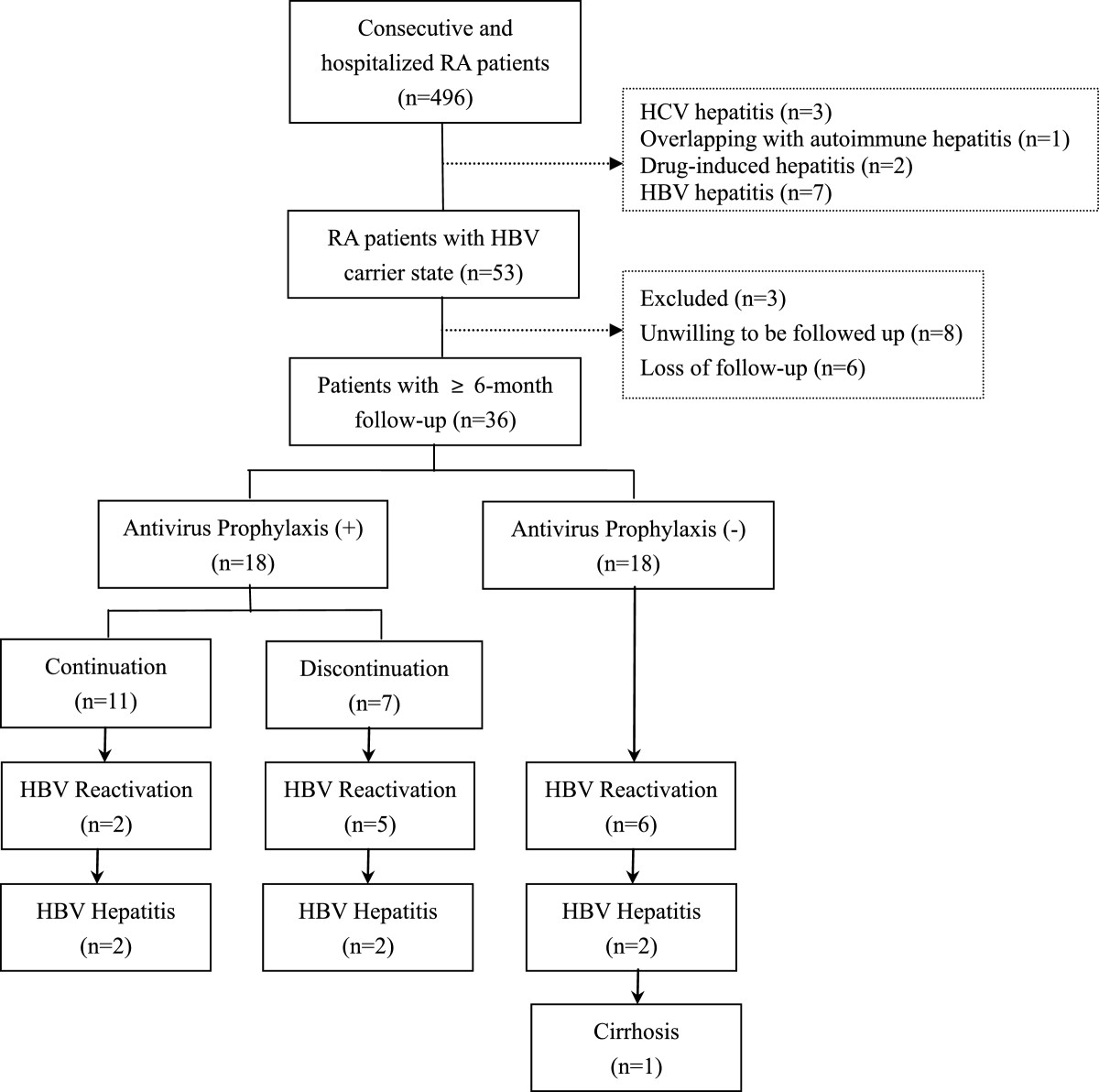
**Flowchart shows the development of hepatitis B virus (HBV) reactivation in rheumatoid arthritis (RA) patients with HBV carrier state with and without antiviral prophylaxis and who discontinued antiviral prophylaxis.**

Fifty-three RA patients with HBV carrier state were included. Two patients overlapping with systemic lupus erythematosus and one patient combined with lower limbs vasculitis were excluded due to high-dose corticosteroids or different immunosuppressants (e.g. cyclophosphamide). Eight patients were unwilling to be followed up. Six patients lost follow-up due to home migration or change to Chinese herbal therapy. Finally, 36 patients were qualified for statistics (Figure 1).Their baseline characteristics were shown in Table 1. Twenty-six patients (72%) were in moderate to high disease activity according to DAS28-crp. Before enrollment, 24 patients (67%) had never received any DMARD or corticosteroid, while the other 12 patients had received corticosteroid (n = 8), MTX (n = 9), LEF (n = 8), SSZ (n = 4) or HCQ (n = 1).

**Table 1:** **Baseline characteristics and therapeutic regimens during follow-up of 36 RA patients with hepatitis B virus (HBV) carrier state** ^▲^

	All Patients (n = 36)	Baseline serum HBV DNA	
		Undetectable (n = 20)	Detectable (n = 16)
**Demographic characters**			
Age (years)	46 ± 15	45 ± 15	48 ± 16
Female proportion	28 (78%)	14 (70%)	14 (88%)
**Disease status**			
Disease duration (months), median (range)	21 (2 ~ 360)	11 (2 ~ 240)	24 (2 ~ 360)
Past history of hepatitis	9 (25%)	5 (25%)	4 (25%)
DAS28-crp, mean ± SD (range)	4.2 ± 1.6 (1.2 ~ 7.6)	4.4 ± 1.7 (1.2 ~ 7.4)	3.9 ± 1.6 (1.7 ~ 7.6)
RF positive rate	21 (58%)	11 (55%)	10 (63%)
Anti-CCP antibody positive rate	17 (47%)	12 (60%)	5 (31%)
CRP (mg/L)	25 ± 33	28 ± 35	20 ± 32
ESR (mm/1 h)	49 ± 43	55 ± 48	40 ± 35
ALT (U/L)	19 ± 10	17 ± 12	21 ± 6^#^
Total bilirubin (μmol/L)	8 ± 4	8 ± 2	11 ± 5
**Therapeutic regimens during follow-up**			
MTX	3 (8%)	2 (10%)	1 (6%)
LEF*	2 (6%)	2 (10%)	0 (0%)
HCQ	1 (3%)	0 (0%)	1 (6%)
MTX + LEF	5 (14%)	3 (15%)	2 (13%)
MTX + SSZ	3 (8%)	2 (10%)	1 (6%)
MTX + HCQ	10 (28%)	4 (20%)	6 (38%)
MTX + HCQ + SSZ	12 (33%)	7 (35%)	5 (31%)
Low-dose Corticosteroid	29 (81%)	15 (75%)	14 (88%)
TNF-α antagonist^△^	4 (11%)	4 (20%)	0

^▲^Data were described with mean ± standard deviation (SD) or number (precentage) unless stated otherwise. DAS28-crp = Disease Activity Score with 28-joint counts modified by CRP; RF = rheumatoid factor; anti-CCP = anti-cyclic citrullinated peptide antibody; CRP = C-reactive protein; ESR = erythrocyte sedimentation rate; ALT = alanine aminotransferase; MTX = methotrexate; LEF = leflunomide; SSZ = sulfasalazine; HCQ = hydroxychloroquine; NSAIDs = non-steroidal anti-inflammatory drugs; TNF = tumor necrosis factor.

*Patients with MTX intolerance due to gastrointestinal discomfort.

△recombinant human TNF-аreceptor: IgG Fc fusion protein (50 mg/w) was given to 2 patients for 4 weeks and infliximab was given to the other 2 patients at a dose of 200 mg for 3 times.

^#^Compared to patients with undetectable baseline HBV DNA, P < 0.05.

Serum HBV-DNA was undetectable (<10^3^copies/mL) in 20 patients and the median of serum HBV-DNA in the other 16 patients was 4.2 × 10^4^ copies/mL (range, 1 × 10^3^ ~ 1.5 × 10^8^). Positive HBeAg was detected in 2 patients with undetectable HBV-DNA and 5 patients with detectable HBV-DNA. There was no significant difference of baseline characteristics between patients with undetectable and detectable HBV-DNA, except that ALT was significantly higher in the latter than in the former (P < 0.05, Table 1) and both ALT were not exceeding normal range.

### HBV reactivation in RA patients with HBV carrier state

Antiviral prophylaxis was recommended for all RA patients with HBV carrier state, but only 18 patients (50%) accepted. Entecavir and tenofovir were recommended to these patients. However, only lamivudine was accepted for economic reason. Sixty-three percentage (10/16) of patients with detectable baseline HBV-DNA accepted antiviral prophylaxis, which tended to be higher than 40% (8/20) of patients with undetectable baseline HBV-DNA (Table 2), but no statistical significance between these two groups was found. Three of seven patients with positive HBeAg accepted antiviral prophylaxis. The median follow-up period was 17.5 months (range, 12 ~ 70 months). HBV-DNA and HBV serological markers of each included patient both at baseline and at the end of follow-up were shown in Additional file 1.

**Table 2:** **Antiviral prophylaxis, HBV reactivation and HBV hepatitis between RA patients with undetectable and detectable baseline HBV-DNA** ^**△**^

Baseline HBV DNA	Antiviral prophylaxis			HBV reactivation	HBV hepatitis
	Yes*	Discontinuation	No		
**Undetectable (n = 20)**	4 (20%)	4 (20%)	12 (60%)	8 (40%)	2 (10%)
**Detectable (n = 16)**	7 (44%)	3 (19%)	6 (37%)	5 (31%)	4 (25%)

*Patients who discontinued antiviral prophylaxis were not included.

△all P > 0.05.

Thirty-six percentage (13/36) of RA patients with HBV carrier state developed HBV reactivation during DMARDs therapy and 17% (6/36) developed HBV hepatitis together with reactivation. Among patients with undetectable baseline HBV-DNA, 40% (8/20) had a switch of serum HBV-DNA from undetectable to detectable. Among patients with detectable baseline HBV-DNA, 31% (5/16) had a 10-fold rise in serum HBV-DNA (Table 2). There was no significant difference in the prevalence of HBV reactivation between the above two groups of patients (p > 0.05, Table 2). Clinical, serological and virological characteristics of all 13 RA patients with HBV reactivation were shown in Table 3. None of them accompanied with HBeAg seroconversion from negative to positive.

**Table 3:** **Clinical, serological and virological characteristics in 13 rheumatoid arthritis (RA) patients with HBV reactivation during immunosuppressive therapy** ^**▲**^

Characteristics	Patient 1	Patient 2	Patient 3	Patient 4	Patient 5	Patient 6	Patient 7	Patient 8	Patient 9	Patient 10	Patient 11	Patient 12	Patient 13
Age/Gender	53/M	39/F	42/F	54/F	71/F	26/F	45/F	55/F	36/F	54/M	17/F	21/M	51/F
Past history of hepatitis	no	yes	no	no	no	yes	no	no	no	yes	yes	yes	yes
HBeAg, baseline/reactivation	-/-	-/-	-/-	-/-	-/-	-/-	+/+	-/-	-/-	-/-	+/+	+/+	-/-
Viral loads at baseline (Copies/mL)	<10^3^	<10^3^	<10^3^	1.0 × 10^3^	<10^3^	<10^3^	<10^3^	<10^3^	7.03 × 10^3^	1.22 × 10^3^	3.18 × 10^5^	<10^3^	1.0 × 10^3^
Viral loads at reactivation (Copies/mL)	3.64 × 10^5^	7.87 × 10^3^	1.92 × 10^3^	2.47 × 10^4^	6.98 × 10^3^	1.15 × 10^4^	3.01 × 10^3^	4.01 × 10^3^	9.91 × 10^5^	1.41 × 10^7^	1.78 × 10^8^	5.26 × 10^7^	1.59 × 10^7^
ALT at baseline (U/L)	8	8	20	19	10	6	15	15	11	30	17	20	27
ALT at reactivation (U/L)	26	20	39	24	26	8	16	123	103	103	825	1880	1274
**Therapeutic regimens during follow-up**													
Corticosteroid	7.5-10 mg/d	no	no	2.5-10 mg/d	5-10 mg/d	10 mg/d	10 mg/d	2.5-10 mg/d	10 mg/d	7.5-10 mg/d	7.5-10 mg/d	no	5-7.5 mg/d
DMARDs	MTX + LEF	MTX + HCQ + SSZ	MTX + LEF	MTX + HCQ	MTX + SSZ	LEF	MTX + HCQ + SSZ	MTX + LEF	MTX + HCQ + SSZ	MTX + HCQ	MTX + LEF	LEF	MTX + HCQ + SSZ
Antiviral prophylaxis	no	Dis	no	Dis	Dis	no	no	no	LAM	LAM	Dis	Dis	no
Time to reactivation (months)	22	24	26	15	10	18	3	22	8	14	5	6	25
Follow-up period (months)	33	43	34	25	22	35	6	52	10	18	11	7	25
HBV hepatitis	no	no	no	no	no	no	no	Anicteric	Anicteric	Anicteric	Icteric	Icteric	Icteric, cirrhosis
**Treatment adjustment after HBV reactivation**													
Adjustments in DMARDs	MTX + HCQ + SSZ	no	no	no	no	no	no	HCQ	MTX + HCQ + SSZ	HCQ	SSZ	HCQ	withdrawal
Antiviral drugs	LAM	no	no	no	no	no	no	LAM	Adefovir dipivoxil	telbivudine	LAM	entecavir	telbivudine

^▲^The sequences of patients were numbered according to the date of HBV reactivation.

HBV = Hepatitis B virus; F = female; M = male; HBeAg = antigen e of HBV; ALT = alanine aminotransferase; DMARDs = disease-modifying antirheumatic drugs; MTX = methrotrexate; LEF = Leflunomide; HCQ = hydroxychloroquine; SSZ = sulfasalazine.

Dis = Discontinuation; LAM = lamivudine.

### Risk factors of HBV reactivation in RA patients with HBV carrier state

Therapeutic regimens for RA during follow-up were shown in Table 1. Low-dose MTX (≤15 mg/w) or MTX + LEF therapy was prescribed for patients responding insufficiently to the original DMARD(s) therapy, intolerant to other DMARDs, in moderate to high disease activity or with prognostically unfavourable factors. Four of 5 patients (80%) taking MTX + LEF developed HBV reactivation, which was significantly higher than 21% (6/28) in patients taking MTX alone or other MTX-based DMARD combinations, p = 0.021, Fisher’s Exact Test. Four patients have received TNF-α antagonist for 4 ~ 6 weeks. After receiving TNF-α receptor: IgG Fc fusion protein (50 mg/w) for 4 weeks, one of two patients changed to MTX + LEF therapy for economic reason and developed HBV reactivation 21 months later (Patient 1 in Table 3); and the other changed to MTX + HCQ + SSZ + Lamivudine therapy and kept undetectable HBV-DNA and normal ALT. After receiving infliximab for 3 times, one of two patients changed to MTX + HCQ + SSZ therapy and developed HBV reactivation 22 months later (Patient 2 in Table 3); and the other changed to MTX + LEF therapy and developed HBV reactivation 24 months later (Patient 3 in Table 3).

**Figure 2:**
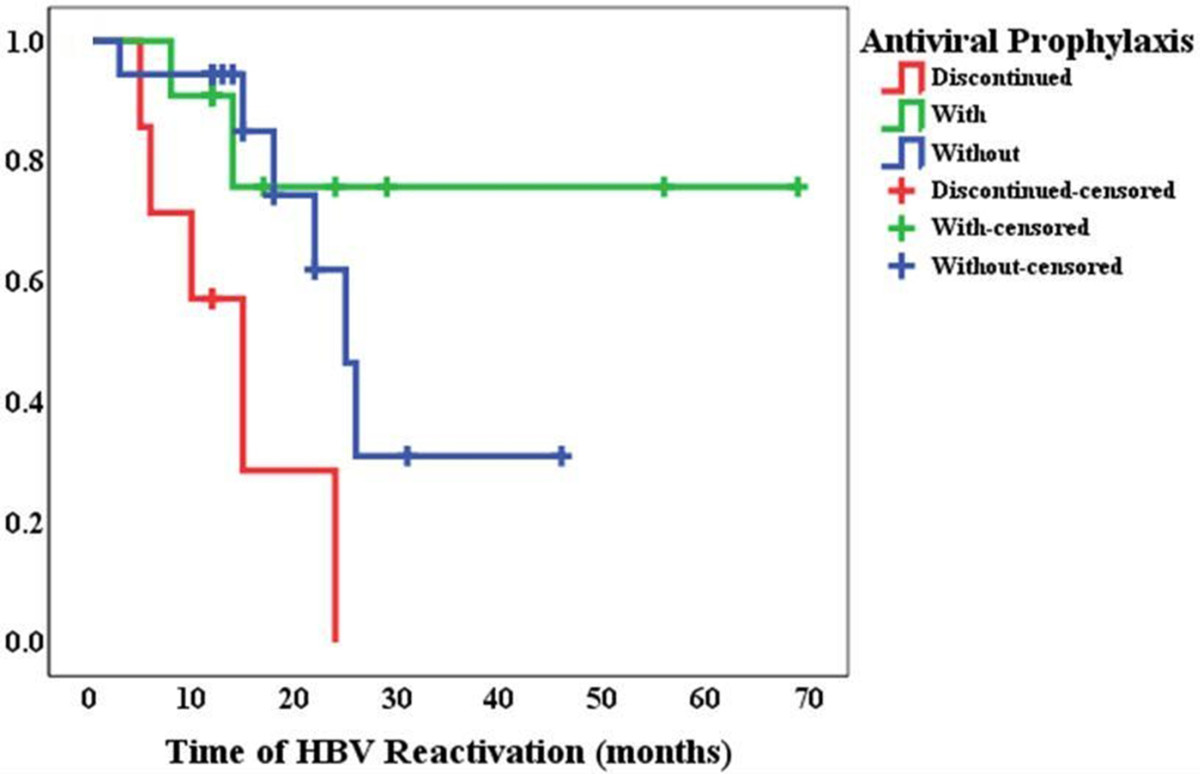
**Survival curve of hepatitis B virus (HBV) reactivation in patients with (green) or without antiviral prophylaxis (blue) or those who discontinued antiviral prophylaxis (red) during immunosuppressive therapy.**

Seven of 18 patients discontinued lamivudine 1 ~ 7 months later by themselves due to high drug cost and 71% of them (5/7) developed HBV reactivation 3 ~ 21 months after discontinuation of lamivudine (Table 3). The prevalence of HBV reactivation in patients who discontinued antiviral prophylaxis was 71% (5/7), which tended to be higher than 33% (6/18) in patients without antiviral prophylaxis or 18% (2/11) in patients with continuous antiviral prophylaxis (Figure 1), although no significant difference was found perhaps due to small sample size (p = 0.122). Notably, survival curve showed the median occurrence time of HBV reactivation in patients who discontinued antiviral prophylaxis was 10 months (95%CI: 1.7 ~ 18 months), which was earlier than that in patients without antiviral prophylaxis, 25 months (95%CI: 20 ~ 30 months) (χ^2^ = 10.754, p = 0.005, Figure 2). Two patients with baseline HBV-DNA of 7.03 × 10^3^copies/mL or 1.22 × 10^3^copies/mL developed HBV reactivation 8 or 14 months later although continuous antiviral prophylaxis with lamivudine (Patient 9 and 10 in Table 3).

Logistic regression analysis was performed on variables including age, past history of hepatitis, baseline HBeAg, baseline load of HBV-DNA, immunosuppressants (such as corticosteroid, MTX, LEF, SSZ, HCQ) and antiviral prophylaxis. Discontinuation of antiviral prophylaxis (OR: 66, p = 0.027), LEF (OR: 64, p = 0.011) and past history of hepatitis (OR: 56, p = 0.013) were risk factors of HBV reactivation for RA patients with HBV carrier state during DMARDs therapy (Table 4). Further analysis showed patients with past history of hepatitis had a higher prevalence of HBV reactivation than those without, 78% (7/9) vs. 22% (6/27), p = 0.005, Fisher’s Exact Test.

**Table 4:** **Step-forward logistic regression analysis on the risk factors of HBV reactivation and HBV hepatitis**

	Coefficient	Standard error	Wald χ^2^	P	Odds ratio	95% CI	
						Lower	Upper
**Risk factors of HBV reactivation**							
Leflunomide	4.2	1.6	6.5	0.011	64	2.6	1591
Discontinuation of antiviral prophylaxis	4.2	1.9	4.9	0.027	66	1.6	2675
Past history of hepatitis	4.0	1.6	6.1	0.013	56	2.3	1355
Constant	-4.2	1.6	6.5	0.011	0.016		
**Risk factor of HBV hepatitis**							
Past history of hepatitis	2.3	1.0	5.4	0.021	10	1.4	70
Constant	-2.5	0.7	11.8	0.001	0.08		

### HBV hepatitis after HBV reactivation in RA patients with HBV carrier state

Among 13 RA patients with HBV reactivation, 6 patients (46%) developed HBV hepatitis together with reactivation. Three patients (23%) developed icteric hepatitis and all of these three patients had past history of icteric hepatitis. Logistic regression analysis on the above variables showed past history of hepatitis was also risk factor of HBV hepatitis after HBV reactivation (OR: 10, p = 0.021, Table 4). Further analysis showed patients with past history of hepatitis had a higher prevalence of HBV hepatitis following reactivation than those without, 44% (4/9) vs 7% (2/27), p = 0.024, Fisher’s Exact Test.

The prevalence of HBV hepatitis in patients with HBV-DNA ≥ 10^5^ copies/mL at reactivation was 83% (5/6), which was significantly higher than 14% (1/7) in patients with HBV-DNA < 10^5^ copies/mL at reactivation (p = 0.029).

### Treatment adjustment after HBV reactivation

Six of 7 patients with HBV-DNA < 10^5^ copies/mL at reactivation (patient 2 ~ 7 in Table 3) were followed up closely without antiviral therapy or DMARDs adjustment and their HBV-DNA returned to undetectable 8 ~ 19 months later. The other one patient with HBV hepatitis although HBV-DNA < 10^5^ copies/mL at reactivation (patient 8 in Table 3) was treated with lamivudine together with HCQ instead of MTX + LEF. Her serum ALT returned to normal and serum HBV-DNA returned to undetectable two months later. However, with relapsed disease activity (DAS28 = 3.6), she was then treated with MTX + HCQ and reached low disease activity 2 months later.

Treatment adjustment of DMARDs for 6 patients with HBV-DNA ≥ 10^5^ copies/mL at reactivation was shown in Table 3. Patient 1 and patient 13 who have not accepted antiviral prophylaxis were prescribed antiviral therapy, and patient 1 chose lamivudine for economic reason. Patient 11 and 12 who have discontinued antiviral prophylaxis was prescribed antiviral therapy, and patient 11 chose lamivudine for economic reason. Patient 9 and 10 who have chosen lamivudine as antiviral prophylaxis were changed to adefovir dipivoxil and telbivudine, respectively. HBV-DNA of patient 1 returned to undetectable 11 months later. Serum ALT of patient 9 ~ 12 returned to normal and serum HBV-DNA decreased 2 ~ 3 months later (still in follow-up). Patient 13 stopped all DMARDs after developing icteric hepatitis and decompensate cirrhosis.

## Discussion

This study provided follow-up records of 36 RA patients with HBV carrier state ranging 12 ~ 70 months in real-world clinical practice and the prevalence of HBV reactivation for RA patients with HBV carrier state during DMARDs therapy was found as high as 36% and the prevalence of HBV hepatitis together with reactivation was 17%. Our study first highlighted poor patient acceptance and discontinuation of antiviral prophylaxis for Chinese RA patients with HBV carrier state. Using survival curve and multivariate regression analysis, our results demonstrated two innovative risk factors of HBV reactivation for RA patients with HBV carrier state during DMARDs therapy, discontinuation of antiviral prophylaxis and past history of hepatitis.

DMARDs can cause unopposed HBV replication or reactivation in liver by suppressing host immune response (e.g. HBV-specific cytotoxic T lymphocyte) [11]. Therefore, HBV reactivation is described mainly on virological terms about serum HBV-DNA with a 10-fold rise or switching from undetectable to detectable. HBeAg seroconversion from negative to positive is also a definition of HBV reactivation. However, our result showed that none of 10 patients with HBV reactivation and negative HBeAg had HBeAg seroconversion. Abolishing or downregulating the production of HBeAg may be partly explained by mutations of the precore or basal core prompter [12]. Detection of serum HBV-DNA may be more important than HBeAg while monitoring the occurrence of HBV reactivation.

Clinical manifestations of HBV reactivation were attributed to elevated HBV-DNA replication and liver injury. Elevated HBV-DNA replication, although asymptomatic sometimes, can induce random integration of HBV-DNA into the host DNA in hepatocytes [6], leading to increased risk of hepatocarcinoma and decreased likelihood of HBV eradication. Liver injury manifests as elevated ALT with or without jaundice. Repeated high ALT peaks with failure to suppress HBV replication were shown to predict higher rates of cirrhosis [13]. Our results showed nearly half of RA patients with HBV reactivation developed HBV hepatitis and one patient developed decompensate cirrhosis, indicating poor outcomes of HBV reactivation for RA patients with HBV carrier state during DMARDs therapy.

### Poor patient acceptance and discontinuation of antiviral prophylaxis should not be ignored for Chinese RA patients with HBV carrier state

Antiviral prophylaxis was recommended by ACR for RA patients with chronic HBV infection during DMARD treatment [8, 9]. Entecavir and tenofovir, which are nucleotide analogs with high antiviral potency and a high barrier to resistance, are both recommended as first line antiviral drugs for patients who have a high HBV-DNA level and/or may receive a lengthy and repeated cycles of immunosuppression (e.g. long-term DMARDs therapy) by European [7], American [6] and Asian-Pacific [5] associations for the study of liver disease. However, in this real-world study, although all patients were recommended entecavir or tenofovir, only 50% of patients accepted lamivudine as antiviral prophylaxis. The most important reason was the cost of antiviral drug, which is much more expensive than conventional DMARDs causing high economic burden for self-paid RA patients, not only in developing countries but also in developed countries [14]. Lamivudine resistance (up to 70% in 5 years) usually occurs after 6–9 months of lamivudine therapy and is responsible for virus breakthrough [5]. Lamivudine resistance partly correlates with YMDD mutation, which develops in 15% ~ 30% per year of HBV patients on lamivudine prophylaxis [15]. Our results showed two patients with continuous lamivudine prophylaxis developed HBV reactivation and hepatitis 8 or 14 months later, with regret that YMDD motif mutation of HBV was not tested. Ryu *et al*. [16] reported one case of HBV reactivation occurred due to YMDD mutation after long-term use of lamivudine in a retrospective study included 15 patients received biological DMARDs under lamivudine prophylaxis.

Withdrawal of antiviral drug usually resulted in rebound of HBV-DNA, accompanying with “flare” of ALT in 19 ~ 50% of patients [17]. Our study showed 71% of patients who discontinued antiviral prophylaxis developed HBV reactivation 3 ~ 21 months after discontinuation. Both survival curve and regression analysis proved that discontinuation of antiviral prophylaxis was risk factor of HBV reactivation for RA patients with HBV carrier state during DMARDs therapy. Therefore, discontinuation of antiviral prophylaxis may be important reason for high prevalence of HBV reactivation in our observational study. Thus, antiviral prophylaxis should be insisted once begun and continued minimal 6 ~ 12 months after suspension of immunosuppressant. Rheumatologists should choose antiviral drugs according to guidelines and informing patients the serious consequences of antiviral drugs discontinuation at each interview.

### Low-dose MTX may be alternative for RA patients with HBV carrier state to control RA disease activity, while LEF should be contraindicated

Not only the risk of HBV reactivation but also the effect of DMARDs on disease activity should be considered when choosing DMARDs for RA patients with HBV carrier state. HCQ, minocycline, SSZ or SSZ + HCQ which can be used for RA patients with HBV carrier state according to guidelines [8, 9] was sometimes inadequate to control moderate to high disease activity, while biologic DMARDs are usually expensive for self-paid patients. Although contraindicated by guidelines [8] MTX and/or LEF sometimes have to be used for controlling RA disease activity. One patient in our study had repeated relapse of RA disease activity when taking HCQ alone after HBV reactivation and HBV hepatitis. After low-dose MTX was added with HCQ, her disease activity was controlled to low disease activity.

In this real-world study, 72% of RA patients had moderate to high disease activity and MTX-based DMARD combination therapy had to be used. Our study showed the prevalence of HBV reactivation in patients taking MTX + LEF was 80%, which was significantly higher than 21% in patients taking MTX alone or other MTX-based DMARD combination therapy. Further regression analysis showed LEF, but not MTX, was risk factor of DMARD-induced HBV reactivation. It was reported that LEF probably activated HBV replication by a nucleotide-reduction–associated increase of mitogen-activated protein kinase (MAPK) p38 phosphorylation in vitro [18]. LEF is also a hepatotoxic drug causing transient elevation of serum ALT in more than 5% of patients [19]. Therefore, LEF should be contraindicated for RA patients with HBV carrier state, while low-dose MTX may be alternative to control moderate to high RA disease activity. One literature review supported this assumption and showed most cases were treated with MTX as a preferred choice [20]. However, 21% of patients taking MTX+ non-LEF DMARDs combination therapy developed HBV reactivation in our study and there are sporadic case reports on MTX-induced HBV reactivation [21–23]. Further prospective cohort study is required to explore the safety and effective of low-dose MTX+ non-LEF DMARDs combination therapy with antiviral therapy for RA patients with HBV carrier state.

Additionally, corticosteroid might promote HBV replication by activating glucocorticoid responsive elements (GRE) on HBV and the risk of high-dose corticosteroid on HBV reactivation in oncology-chemotherapy patients has been comfirmed [11]. Other than patients with hematology-oncology diseases or some other rheumatic diseases, low-dose glucocorticoids should be considered as part of the initial treatment strategy (in combination with one or more conventional DMARDs) for RA patients for up to 6 months, but should be tapered as rapidly as clinically feasible according to 2013 EULAR recommendations [24]. One small-scale prospective study showed coadministration of low-dose corticosteroid had a significant correlation with HBV reactivation among RA patients with HBV carrier state without antiviral prophylaxis [25]. Our study did not find corticosteroid as a risk factor of HBV reactivation for RA patients with HBV carrier state during DMARDs therapy. Since our study included more patients (81%) taking low-dose corticosteroid, further study with larger sample size is needed to evaluate the exact risk of low-dose corticosteroid on HBV reactivation for RA patients with HBV carrier state during DMARDs therapy.

One of limitations in this study was lack of data about biologic DMARDs, since only 4 patients receiving TNF-α antagonist for 4 ~ 6 weeks were included. HBV reactivation occurred 21 ~ 24 months after discontinuation of TNF-α antagonist in 3 patients, which might be attributed to subsequent conventional DMARDs, rather than TNF-α antagonist. Recently, Costa *et al*. [26] reported long-term use of TNF-α antagonist alone was safe for 15 psoriatic arthritis patients with chronic hepatitis C virus (HCV) infection in the absence of specific therapy for HCV. Giannitti *et al*. [27] reported tocilizumab combined with cyclosporine-A was effective and safe for a RA patient with chronic HCV infection. Cyclosporine-A could control HCV replication by inhibition of cyclophilin-B (while the inhibition of calcineurin causes immunosuppressive effect) and may be safe in patients with autoimmune disorders and concomitant HCV infection [28]. However, litter is known about the safety of cyclosporine-A in patients with RA and concomitant HBV infection. Additionally, RA patients who were HBsAg-negative anti-HBc-positive potential occult carriers [29] should also be emphasized.

## Conclusions

The results of our study suggest that poor patient acceptance and discontinuation of antiviral prophylaxis should not be ignored for Chinese RA patients with HBV carrier state in real-world clinical practice. Discontinuation of antiviral prophylaxis, past history of hepatitis and LEF might increase risk of HBV reactivation for RA patients with HBV carrier state during DMARDs therapy.

## Electronic supplementary material

Additional file 1:HBV serological markers for each patient both at baseline and at the end of follow-up*.(PDF 61 KB)

Below are the links to the authors’ original submitted files for images.

Authors’ original file for figure 1

Authors’ original file for figure 2

## Abbreviations

HBV: Hepatitis B Virus
HBsAg: Surface Antigen of HBV
ALT: Alanine Amino-Transferase
RA: Rheumatoid Arthritis
DMARDs: Disease Modifying Antirheumatic Drug
ACR: American College of Rheumatology
HCQ: Hydroxychloroquine
MTX: Methotrexate
LEF: Leflunomide
EULAR: European League Against Rheumatism
TBiL: Total Billirubin
HBsAb: Antibody to HBsAg
HBeAg: E Antigen of HBV
HBeAb: Antibody to HBeAg
HBcAb: Antibody to Core Antigen of HBV
OR: Odds ratio
CI: Confidence interval
MAPK: Mitogen-Activated Protein Kinase
GRE: Glucocorticoid rresponsive elements
HCV: Hepatitis C virus.
