# Supplementary material for: Discontinuation of antiviral prophylaxis correlates with high prevalence of hepatitis B virus (HBV) reactivation in rheumatoid arthritis patients with HBV carrier state: a real-world clinical practice
Source: BMC Musculoskelet Disord. 2014 Dec 22;15:449. doi: 10.1186/1471-2474-15-449 (PMC4320507; doi:10.1186/1471-2474-15-449)
Supplement: Supplementary file 1 — Additional file 1:HBV serological markers for each patient both at baseline and at the end of follow-up*.(PDF 61 KB) [file 12891_2014_2397_MOESM1_ESM.pdf]

Additional file 1. HBV serological markers for each patient both at baseline and at the end of follow-up*

Characteristics	Patient 1	Patient 2	Patient 3	Patient 4	Patient 5	Patient 6	Patient 7	Patient 8	Patient 9	Patient 10	Patient 11	Patient 12	Patient 13
HBsAg, baseline/ at reactivation	+/+	+/+	+/+	+/+	+/+	+/+	+/+	+/+	+/+	+/+	+/+	+/+	+/+
HBsAb, baseline/ at reactivation	-/-	-/-	-/-	-/-	-/-	-/-	-/-	-/-	-/-	-/-	-/-	-/-	-/-
HBeAg, baseline/ at reactivation	-/-	-/-	-/-	-/-	-/-	-/-	+/+	-/-	-/-	-/-	+/+	+/+	-/-
HBeAb, baseline/ at reactivation	+/+	+/+	+/+	+/+	+/+	+/+	-/-	+/+	+/+	+/+	-/-	-/-	+/+
HBcAb, baseline/ at reactivation	+/+	+/+	+/+	+/+	+/+	+/+	+/+	+/+	+/+	+/+	+/+	+/+	+/+
Viral loads at baseline (Copies/mL)	$<10^3$	$<10^3$	$<10^3$	1.0×10^3	$<10^3$	$<10^3$	$<10^3$	$<10^3$	7.03×10^3	1.22×10^3	3.18×10^5	$<10^3$	1.0×10^3
Viral loads at reactivation (Copies/mL)	3.64×10^5	7.87×10^3	1.92×10^3	2.47×10^4	6.98×10^3	1.15×10^4	3.01×10^3	4.01×10^3	9.91×10^5	1.41×10^7	1.78×10^8	5.26×10^7	1.59×10^7

Characteristics	Patient 14	Patient 15	Patient 16	Patient 17	Patient 18	Patient 19	Patient 20	Patient 21	Patient 22	Patient 23	Patient 24	Patient 25
HBsAg, baseline/ at the end of follow-up	+/+	+/+	+/+	+/+	+/+	+/+	+/+	+/+	+/+	+/+	+/+	+/+
HBsAb, baseline/ at the end of follow-up	-/-	-/-	-/-	-/-	-/-	-/-	-/-	-/-	-/-	-/-	-/-	-/-
HBeAg, baseline/ at the end of follow-up	-/-	-/-	-/-	-/-	-/-	-/-	-/-	-/-	-/-	-/-	-/-	-/-
HBeAb, baseline/ at the end of follow-up	+/+	+/+	+/+	+/+	+/+	+/+	+/+	+/+	+/+	+/+	+/+	+/+
HBcAb, baseline/ at the end of follow-up	+/+	+/+	+/+	+/+	+/+	+/+	+/+	+/+	+/+	+/+	+/+	+/+
Viral loads at baseline (Copies/mL)	$<10^3$	$<10^3$	$<10^3$	$<10^3$	$<10^3$	$<10^3$	$<10^3$	$<10^3$	$<10^3$	$<10^3$	$<10^3$	$<10^3$
Viral loads at the end of follow-up (Copies/mL)	$<10^3$	$<10^3$	$<10^3$	$<10^3$	$<10^3$	$<10^3$	$<10^3$	$<10^3$	$<10^3$	$<10^3$	$<10^3$	$<10^3$

Characteristics	Patient 26	Patient 27	Patient 28	Patient 29	Patient 30	Patient 31	Patient 32	Patient 33	Patient 34	Patient 35	Patient 36
HBsAg, baseline/ at the end of follow-up	+/+	+/+	+/+	+/+	+/+	+/+	+/+	+/+	+/+	+/+	+/+
HBsAb, baseline/ at the end of follow-up	-/-	-/-	-/-	-/-	-/-	-/-	-/-	-/-	-/-	-/-	-/-
HBeAg, baseline/ at the end of follow-up	+/+	+/+	+/+	-/-	-/-	+/+	-/-	-/-	-/-	-/-	-/-
HBeAb, baseline/ at the end of follow-up	-/-	-/-	-/-	+/+	+/+	-/-	+/+	+/+	+/+	+/+	+/+
HBcAb, baseline/ at the end of follow-up	+/+	+/+	+/+	+/+	+/+	+/+	+/+	+/+	+/+	+/+	+/+
Viral loads at baseline (Copies/mL)	1.51×10^8	6.06×10^7	8.89×10^6	3.94×10^5	1.71×10^5	1.39×10^5	7.04×10^4	1.33×10^4	6.31×10^3	4.01×10^3	2.0×10^3
Viral loads at the end of follow-up (Copies/mL)	6.99×10^7	5.54×10^5	6.78×10^4	3.54×10^4	$<5.0\times10^2$	6.72×10^4	5.12×10^4	5.0×10^2	4.79×10^3	$<5.0\times10^2$	2.18×10^3

* The major endpoint of this study was HBV reactivation. For patient 1~13 who developed HBV reactivation, “at the end of follow-up” was equal to “at reactivation”.
