# Supplementary figures and images for: Discontinuation of antiviral prophylaxis correlates with high prevalence of hepatitis B virus (HBV) reactivation in rheumatoid arthritis patients with HBV carrier state: a real-world clinical practice
Source: BMC Musculoskelet Disord. 2014 Dec 22;15:449. doi: 10.1186/1471-2474-15-449 (PMC4320507; doi:10.1186/1471-2474-15-449)

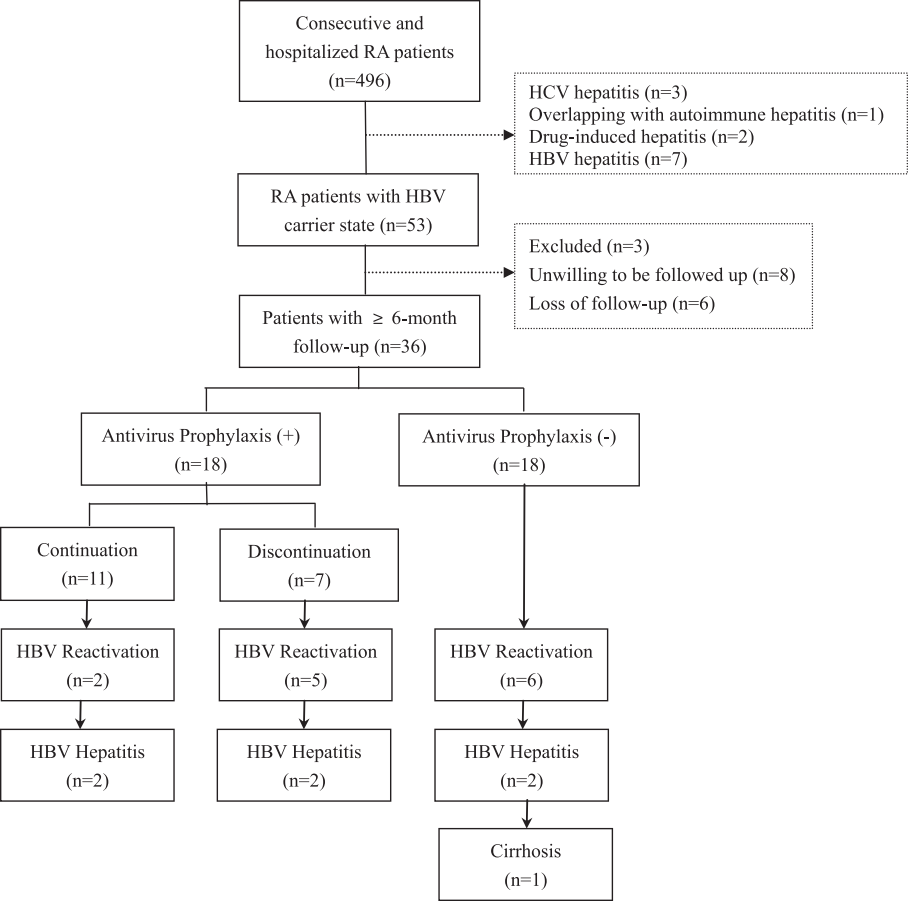

Supplement: Supplementary file 2 — Authors’ original file for figure 1 [file 12891_2014_2397_MOESM2_ESM.pdf]

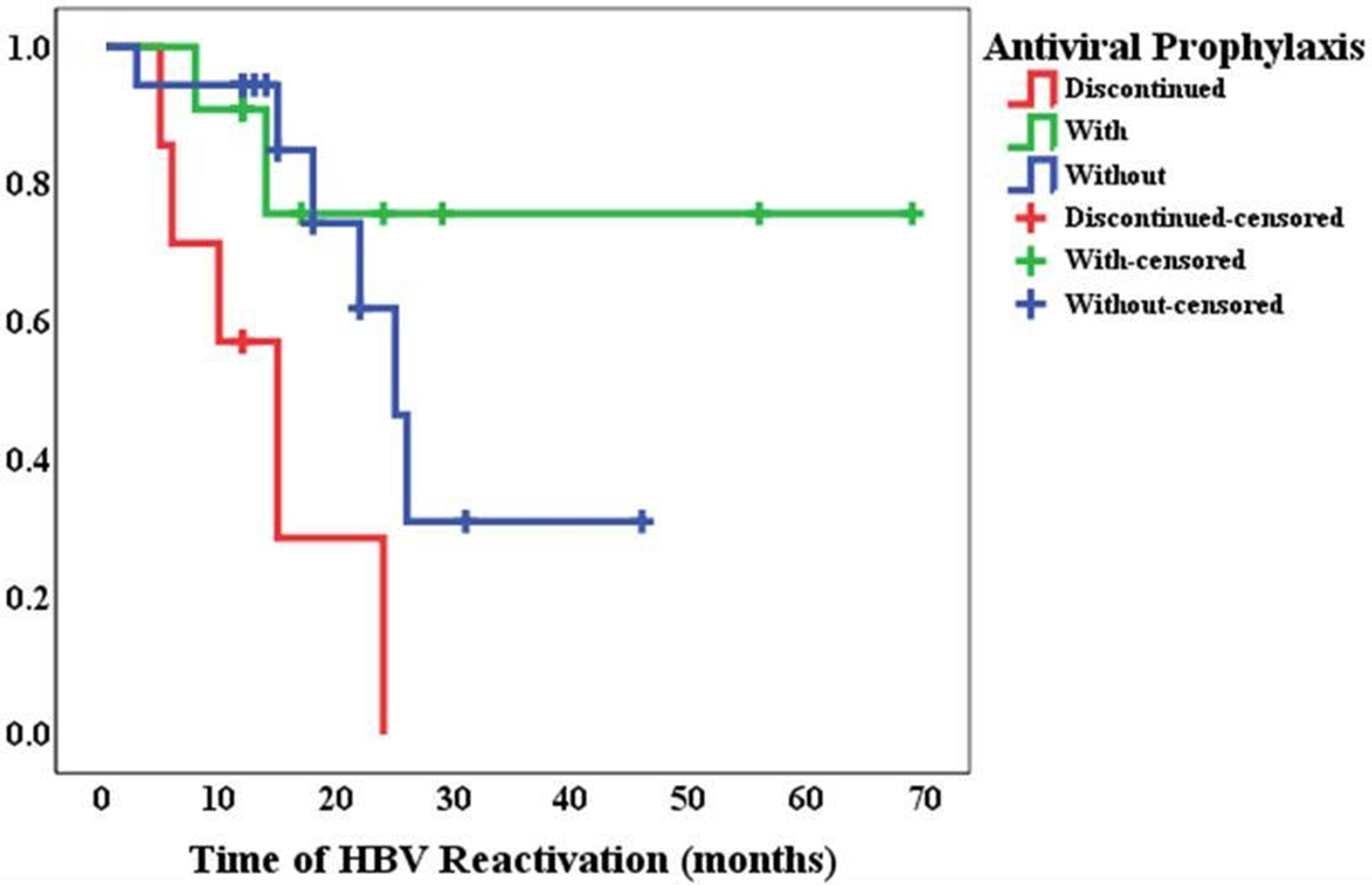

Supplement: Supplementary file 3 — Authors’ original file for figure 2 [file 12891_2014_2397_MOESM3_ESM.tif]
